# Roles of *Tet2* in meiosis, fertility and reproductive aging

**DOI:** 10.1007/s13238-020-00805-8

**Published:** 2020-11-20

**Authors:** Huasong Wang, Linlin Liu, Mo Gou, Guian Huang, Chenglei Tian, Jiao Yang, Haiying Wang, Qin Xu, Guo_Liang Xu, Lin Liu

**Affiliations:** 1 Department of Cell Biology and Genetics, College of Life Sciences, Nankai University, Tianjin, 300071 China; 2 State Key Laboratory of Medicinal Chemical Biology, Nankai University, Tianjin, 300071 China; 3 State Key Laboratory of Molecular Biology, Shanghai Institute of Biochemistry and Cell Biology, Chinese Academy of Sciences, Shanghai, 200031 China; 4 Key Laboratory of Medical Epigenetics, Institutes of Biomedical Sciences, Fudan University, Shanghai, 200032 China

**Dear Editor,**

Ten-eleven translocation (Tet) enzymes play important roles in DNA demethylation involved in various biological processes including stem cell pluripotency and differentiation, and tumorigenesis (Dawlaty et al., 2013; Wu and Zhang, 2017). *Tet2* deficiency or mutation leads to severe hematopoietic defects or myeloid malignancies in mice (Delhommeau et al., 2009; Ko et al., 2010; Moran-Crusio et al., 2011). Restoration of *TET2* blocks aberrant self-renewal and leukemia progression in patients possessing TET2 mutations (Cimmino et al., 2017). DNA methylation-based biomarkers, or “epigenetic clocks”, link developmental and maintenance processes to biological aging (Horvath and Raj, 2018). Interestingly, age-associated *TET2* mutations have been found to drive myeloid dysfunction, cancer and cardiovascular disease (review (Ferrone et al., 2020)). Nevertheless, there has been lack of direct evidence demonstrating that *Tet2* mutations or deficiency can actually accelerate aging.

Reproductive aging particularly in female germline predates the aging of somatic organs in the body. The ovaries play an important role in maintaining the normal function of the female reproductive system and hormone secretion. Ovarian aging is mainly characterized by a sharp decrease in the number of oocytes and follicles as well as declined oocyte quality. Loss of *Tet1* causes meiosis defects in fetal ovaries and in adult testis (Huang et al., 2020; Yamaguchi et al., 2012), and reduces oocyte number (Yamaguchi et al., 2012). Here we show that Tet2 plays multiple roles during meiosis and oocyte development. Moreover, *Tet2* deficiency does not affect oocyte number, unlike *Tet1* (Yamaguchi et al., 2012), but noticeably delays meiotic progression and reduces oocyte quality and mouse fertility, accelerating age-associated infertility.

To study whether Tet2 regulates fertility with age, we employed *Tet2*-deficient mice generated by deleting exon 3 (See methods). Homozygous mutant mice (*Tet2*^−/−^) were generated by crossing the heterozygous mice. The primers were designed to detect three different genotypes (wild-type (WT) *Tet2*^+/+^, *Tet2*^+/−^ and *Tet2*^−/−^) (Fig. S1A), and the exon 3 deletion in *Tet2*^−/−^ female ovaries was also validated by qPCR of mRNA expression (Fig. S1B). Probably Tet2 protein level is very low in adult ovary, such that Tet2 protein signal could not be detectable. Thus, we confirmed that Tet2 protein was absent in *Tet2*^−/−^ blastocysts by immunofluorescence (Fig. S1C). *Tet2*^−/−^ mice were born at normal Mendelian ratio (Fig. S1D). The 5hmC levels were decreased by immunofluorescence in *Tet2*-deficient oocytes and granulosa cells (Fig. S1E and S1F). Hence, *Tet2* knockout decreases the 5hmC level, as expected.

*Tet2*^−/−^ female mice at young, middle-age and old age (specified in the figure legend) after successful mating with young males exhibited reduced fecundity as shown by smaller litter size, compared with age-matched WT females. The litter size reduced by half when the *Tet2*^−/−^ mice reached at middle-age, and most old *Tet2*^−/−^ mice failed to give birth to a pup (Fig. 1A). Ovarian weight of WT females increased at middle-age and then decreased at the old age. Ovarian weight of *Tet2*^−/−^ mice was generally lighter than that of WT mice at young and middle-age, but did not differ from WT mice at the old age (Fig. S1G and S1H). However, the average number of follicles at various developmental stages in *Tet2*^−/−^ ovaries did not differ from that of WT ovaries regardless of age (Fig. S1I and S1J). Therefore, *Tet2* deficiency decreases fecundity and accelerates infertility with age, but intriguingly, the infertility is not related to follicle reserve or oocyte numbers.

**Figure 1:**
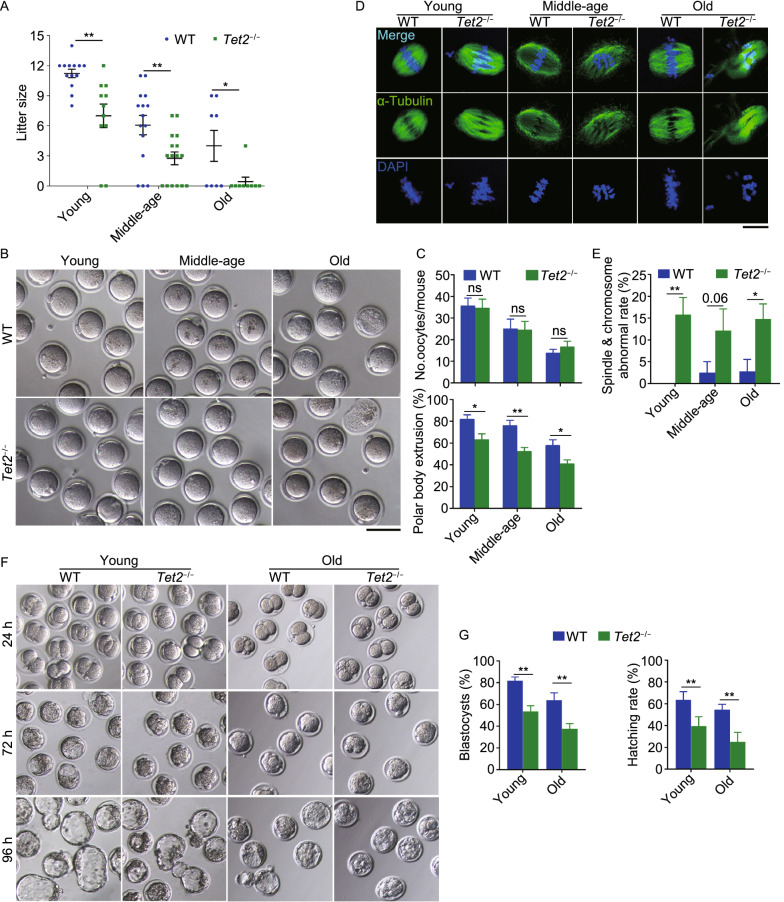
***Tet2*** **knockout causes subfertility and impairs oocyte and early embryo development**. (A) Average litter size of wild-type (WT) and *Tet2* knockout (*Tet2*^−/−^) female mice at three different ages (young, 2–4 months; middle-age, 7–9 months; old, 10–12 months). *n* > 10 for successfully mated female mice for young and middle-age group and *n* ≥ 8 mice for old group are pooled. Each spot in round (blue) or square (green) shape represents the number of pups delivered from a successfully mated female mouse. (B) Representative images of superovulated oocytes from WT and *Tet2*^*−/−*^ female mice at three different ages. Scale bar, 100 μm. (C) Comparison of oocyte yield per mouse (top) and percentage of the first polar body extrusion (bottom). *n* = 4–6 mice per group. (D) Representative images of oocytes stained with α-Tubulin antibody (green) and DAPI (blue) from three different age groups. Scale bar, 10 μm. (E) Spindle and chromosome abnormal rate between WT and *Tet2*^−/−^ oocytes from three different age groups. *n* = 3–4 mice per group. (F) Morphology of embryo development at 2-cell (24 h), morula (72 h) and blastocyst (96 h) stage following *in vitro* fertilization of oocytes. Oocytes were obtained from WT and *Tet2*^*−/−*^ mice from young or old group and sperm from healthy ICR male mice. (G) Percentage of developed blastocysts or blastocyst hatching rate 96 h after *in vitro* fertilization from young and old oocytes. *n* = 3–5 mice, and about 50 blastocysts were counted for each group. The bars indicate mean ± SEM. *P < 0.05; **P < 0.01; ns, no significant difference

We assumed that *Tet2* deficiency may negatively impact germ cell development and oocyte quality. We initially examined whether *Tet2* deficiency affects meiosis in the fetal gonad. Female meiosis undergoes unique long prophase I, including leptotene, zygotene, pachytene and diplotene, followed by arrest at diakinesis during E13.5–E18.5 after PGC specification (Saitou and Miyauchi, 2016). We chose four embryonic days, including E15.5, E16.5, E17.5 and E18.5, to observe meiosis progression. All the four stages in prophase I could be found at the right time in WT meiocytes, but meiosis progression notably was postponed by *Tet2* deficiency (Fig. S2A). While nearly 60% of meiocytes reached pachytene stage at E16.5 in WT meiocytes with clear synapsis indicated by pairing of SYCP3 and SYCP1 (synaptonemal complex protein 3/1), only about 30% of *Tet2*^−/−^ meiocytes entered pachytene and more than 60% remained at zygotene stage (Fig. S2B). At E18.5, 56% of WT meiocytes were at diplotene stage, whereas only 18% reached diplotene and most (73%) still were at pachytene stage. These results demonstrated that *Tet2* is required for completion of normal meiosis progression at right timing.

To understand the molecular basis underlying role of *Tet2* in the initiation of meiosis, we purified germ cells from E13.5 female gonads of *Tet2*^−/−^ and WT, and profiled their transcriptome by RNA-seq. We focused on this time point because this is when female PGCs enter meiotic prophase after epigenetic reprogramming (Saitou and Miyauchi, 2016). Important meiotic genes involving meiosis initiation, synapsis formation, crossover and recombination, including *Mei1*, *Ccnb3*, *Meioc*, *Sycp1*, *Sycp2*, *Msh5*, *Rad21l* and *Prdm9*, were downregulated in *Tet2*^−/−^ compare to WT germ cells (Fig. S2C). The transcription profile partially interpreted delayed meiosis progression in embryonic gonads resulting from *Tet2* deficiency.

The initiating event of meiotic recombination is a programmed DNA double-strand breaks (DSBs) introduced to the genome by Spo11. The DSBs which can be detected by the presence of γH2AX, must be subsequently repaired to form crossovers or noncrossovers. DNA double-strand breaks and repair are required for homologous pairing and synapsis prior to meiotic recombination. Delayed homologous synapsis and meiotic progression might result from defective DNA repair during meiosis. To test this hypothesis, we assayed DNA damage by immunofluorescence assay for γH2AX and other key meiosis-associated proteins in the meiocyte spreads prepared from E17.5 gonads. Proportion of γH2AX positive and partially positive meiocytes increased at pachytene in *Tet2*^−/−^ mice (Fig. S3A–C), suggesting that DSBs are not completely repaired in some *Tet2*^−/−^ meiocytes. RAD51 is a DSB repair-associated recombinase needed for both mitotic and meiotic recombination. Yet, RAD51 foci were not increased in *Tet2*^−/−^, compared to WT meiocytes (Fig. S3D and S3E). Late-recombination marker MLH1 normally appears at the designated crossover sites. Number of MLH1 foci also did not differ between *Tet2*^−/−^ and WT meiocytes (Fig. S3F and S3G), suggesting that the crossover formation was not impaired. SYCP1/SYCP3-positive staining signal was used to indicate synaptonemal complexes, and 20 synaptonemal complex elements could be found per *Tet2*^−/−^ meiocyte, like in WT cell (Fig. S3H and S3I). These results suggest that approximately 60% of *Tet2*^−/−^ meiocytes can still undergo normal homologous chromosome pairing and meiosis recombination at E17.5.

Next, we asked whether DNA damage could accumulate in *Tet2*^−/−^ oocytes compared to that of WT oocytes at later stages. We detected the level of γH2AX in the ovary at postnatal day (PD) 6, because almost all of meiocytes developed to primordial follicles at this time. In primordial follicles, more intense γH2AX signal was found in the nucleus of *Tet2*^−/−^ than in WT oocytes (Fig. S3J and S3K). Elevated DNA damage indicated by γH2AX foci was still present in *Tet2*^−/−^ GV oocytes from young and old female mice (Fig. S3L and S3M). γH2AX staining pattern of WT meiocytes and oocytes in primordial and mature follicles resembled those reported previously (Yamaguchi et al., 2012). These data suggest important role of Tet2 in DNA repair of early germ cells and oocytes.

The quality of mature oocytes is a major determinant of reproductive capacity and fertility of females. We assessed the number and morphology of oocytes collected from mice at different age 16–17 h following superovulation treatment. The number of oocytes decreased with age in WT females, well-recognized phenomenon in reproductive aging, and also in *Tet2*^−/−^ female mice (Fig. 1B and 1C). The number of oocytes obtained from *Tet2*^−/−^ mice did not differ from that of WT females regardless of age, and this corroborated similar number of follicles shown earlier (Fig. S1I and S1J). However, polar body extrusion was notably suppressed or delayed in *Tet2*^−/−^ oocytes and this became more evident with age, unlike WT oocytes (Fig. 1B and 1C). Furthermore, the frequency of abnormal or disrupted spindles and chromosome misalignment at metaphase was increased in *Tet2*^−/−^ oocytes from mice at different age (Fig. 1D and 1E). Consequently, rate of development to blastocysts of *Tet2*^−/−^ oocytes following *in vitro* fertilization (IVF) was lower than that of WT, and further declined with age (Fig. 1F and 1G). While successfully fertilized WT oocytes cleaved and reached blastocysts by 72 h in culture, most of *Tet2*^−/−^ oocytes and oocytes from old WT mice still were at morula stage, indicative of delayed embryo development. In addition, blastocysts developed from *Tet2*^−/−^ oocytes were smaller, and the percentage of hatching blastocysts also was lower at young and old age, compared to that of age-matched WT controls (Fig. 1F and 1G). These data showed that *Tet2*^−/−^ deficiency impairs polar body extrusion, spindle structure and chromosome alignment, reducing oocyte quality and subsequent embryo development.

The spindle assembly checkpoint (SAC) safeguards correct chromosome alignment by sensing unattached kinetochores and delaying anaphase onset (London and Biggins, 2014). Mad2 and Cenpe, as a bridge to connect kinetochore and microtubule, would be removed from kinetochores 8 h post germinal vesicle breakdown (GVBD) (Marangos et al., 2015). We wondered whether the two proteins were implicated in reduced polar body extrusion in *Tet2*^−/−^ oocytes. More strongly stained Mad2 and Cenpe signals at kinetochores were revealed in *Tet2*^−/−^ oocytes 8 h post GVBD, compared to WT oocytes (Fig. S4A–D). Moreover, by careful examination of spindles and chromosome morphology of oocytes 16–17 h after superovulation, we observed apparently more abnormal oocytes (about 60%), which were at MI, anaphase or telophase stage with misaligned chromosomes or disrupted spindles in *Tet2*^−/−^ mice and these abnormalities increased to nearly 80% at old age, whereas a majority (about 80%) of oocytes from young WT mice displayed normal meiosis with appropriate chromosome alignment at the metaphase spindle (Fig. S4E and S4F). The delayed MI progression is linked to postponed or suppressed polar body extrusion. Thus, Tet2 is implicated in the checkpoint control during chromosome congregation and separation.

To understand the molecular bases of the declined reproductive capacity and associated meiotic defects caused by *Tet2* deficiency shown above, we performed transcriptome analysis by single-cell RNA-seq of WT and *Tet2*^−/−^ MII oocytes collected from young and old mice (Fig. 2A). Based on the Principal Component Analysis (PCA), the distributions of two PCA components differed among WT and *Tet2*^−/−^ oocytes at young and old age, and some overlapped between young *Tet2*^−/−^ and old WT oocytes. Distribution of PCA in old *Tet2*^−/−^ oocytes was more complicated, suggesting that *Tet2* deficiency and aging jointly affected the oocyte transcription (Fig. 2A). Differentially expressed genes (DEGs) were obtained by Deseq2 and genes highly expressed in different groups were compared by pheatmap. Genes important for meiosis *Sycp3* and *Dmc1*, *Wapl*, required for accurate meiosis I chromosome segregation and controlling SAC, *Mad2l1*, *Mad1l1* and *Zfp207* for chromosome alignment during meiosis I, and *Fbxw7*, *Ythdf2*, critical for oocyte maturation and competence, were highly expressed in young WT oocytes but downregulated in oocytes from other three groups (Fig. 2B). Moreover, we performed GO and KEGG enrichment using KOBAS database with the adjusted *P*-value (FDR < 0.05, by statistical algorithm of Benjamini and Hochberg). Genes highly expressed in young WT oocytes but downregulated in other three groups were enriched in cellular response to DNA damage stimulus, DNA repair, sister chromatid segregation, meiosis I cell cycle process and meiotic cell cycle (Fig. 2C). KEGG analysis also showed that highly expressed genes in young WT group were enriched in oocyte meiosis, cell cycle, and metabolic pathways. The transcriptome data suggested that *Tet* deficiency reduces DNA repair and meiosis progression and SAC, leading to accelerated decline in oocyte competence with age.

**Figure 2:**
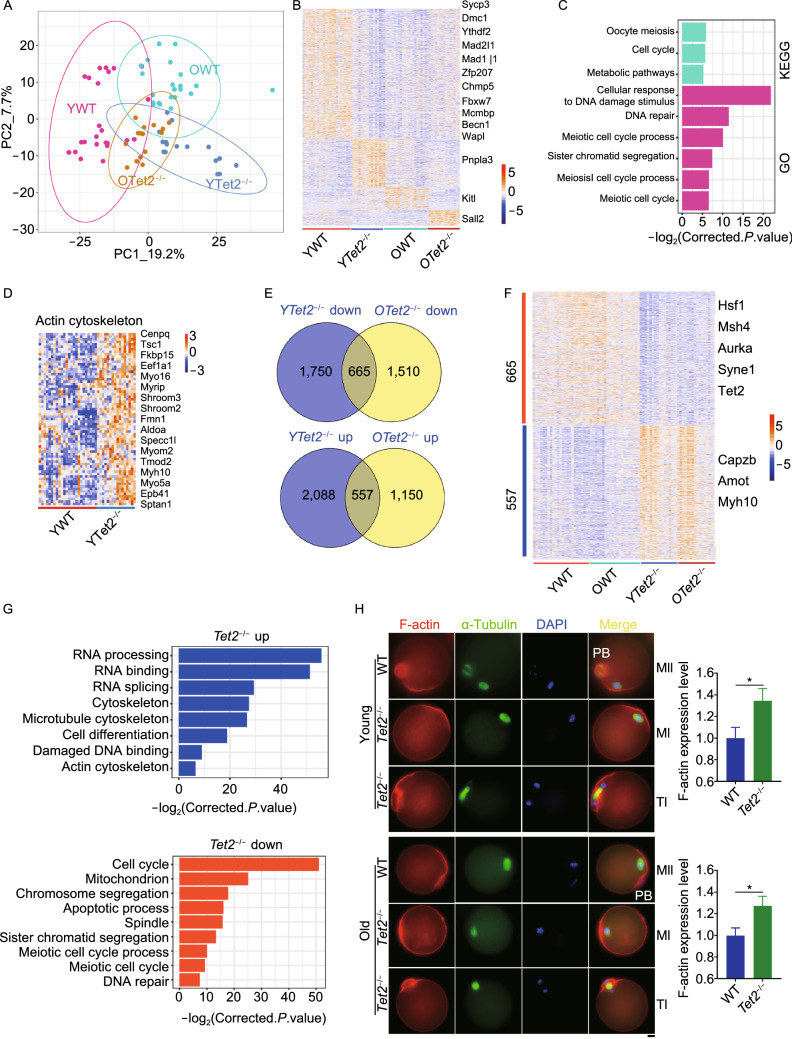
**Differential gene expression of** ***Tet2***^***−/−***^ **and WT oocytes**. (A) PCA analysis of young WT (*n* = 24), *Tet2*^−/−^ (*n* = 16), old WT (*n* = 22) and *Tet2*^−/−^ (*n* = 16) oocytes using the CPM for all genes from 2–3 mice. (B) Pheatmap showing differential gene expression of young WT, *Tet2*^−/−^, old WT and *Tet2*^−/−^ oocytes. (C) Barplot showing the enrichment of genes highly expressed in young WT oocytes but lowly expressed in other groups. X-axis represents the corrected p-value (FDR) using Benjamini and Hochberg. (D) Pheatmap showing genes enriched in actin cytoskeleton after *Tet2* knockout. (E) Venn diagram of upregulated and downregulated genes in young and old *Tet2*^−/−^ oocytes, compared with WT oocytes. (F) Pheatmap showing genes upregulated and downregulated in both *Tet2*^−/−^ young and old oocytes. (G) Bar plot illustrating the enrichment of genes upregulated or downregulated after *Tet2* knockout. X-axis represented the corrected p-value (FDR) using Benjamini and Hochberg. (H) Representative images of oocytes co-stained with F-actin and α-Tubulin by immunofluorescence microscopy. Scale bar, 10 μm. Right panel, Relative expression F-actin level by quantification of immunofluorescence intensity averaged from various oocytes using Image J (*n* = 5–7)

Moreover, we looked at transcriptional changes in oocytes during natural aging, to understand the reduced quality of oocytes. Aging resulted in differential expression of genes, including downregulation of *Id3*, *Sycp3*, *Dmc1*, and *Sycp1*, and upregulation of *Atm*, *Rad50*, and *Brca2* (Fig. S5A). t-SNE showed distribution in the gene expression between oocytes of young and old mice (Fig. S5B). By signaling pathway analysis, genes downregulated were enriched in oocyte meiosis and maturation and regulation of actin cytoskeleton, but genes related to Alzheimer’s disease were upregulated (Fig. S5C and S5D). By functional enrichment (GO) analysis, genes related to methylation were upregulated, but genes involved in reciprocal meiotic recombination, demethylation, sister chromatid cohesion, DNA repair and embryo development were downregulated in old WT oocytes (Fig. S5E and S5F). These molecular defects in DNA repair, oocyte maturation, spindle and sister chromatid cohesion are consistent with declined function of oocytes with age shown previously.

We further analyzed single-cell transcriptome data of *Tet2*^−/−^ and WT oocytes (*n* = 16 and 24, respectively) collected from 2–3 mice at young age. Upregulated genes in *Tet2*^−/−^ oocytes surprisingly were enriched in actin binding and actin cytoskeleton (Fig. 2D). We also compared *Tet2*^−/−^ with WT oocytes from young and old mice. Venn diagram revealed 665 genes co-downregulated and 557 genes co-upregulated after *Tet2* loss (Fig. 2E). As expected, *Tet2* was downregulated in *Tet2*^−/−^ compared with WT oocytes. In addition, genes for spindle assembly checkpoint and DNA repair such as *Hsf1*, *Msh4*, *Aurka*, and *Syne1*, were downregulated while genes related with actin cytoskeleton organization, including *Myh10*, *Captz* and *Amot*, were among the highest upregulated in *Tet2*^−/−^ oocytes (Fig. 2F). Upregulated genes after *Tet2* knock-out also included RNA processing and splicing, cell differentiation, damaged DNA binding and actin cytoskeleton (Fig. 2G). Genes downregulated were enriched in cell cycle, mitochondrion, apoptotic process, spindle, sister chromatid segregation, meiotic cell cycle process and DNA repair (Fig. 2G).

Genes associated with increased DNA damage, aberrant upregulation of actin cytoskeleton and downregulated spindle checkpoint of *Tet2*^−/−^ oocytes got our attention, and these defects together likely link to suppression or delay of polar body extrusion resulting from *Tet2* deficiency. To substantiate the transcriptome results, we performed immunostaining of F-actin filament in oocytes. Indeed, normal MII oocytes of WT mice exhibited polar body extrusion enclosed within actin ring, and well separated chromosomes surrounded by actin filament residue (Fig. 2H). However, stronger actin filament immunofluorescence signal was found in oocytes of *Tet2*^−/−^ mice, compared to WT mice. Distribution of more uniformly stained actin filaments in *Tet2*^−/−^ oocytes coincided with MI arrest or anaphase or telophase (TI) without clear polar body separation, or with delayed polar body extrusion (Fig. 2H). Aberrantly distributed actin at the high expression level likely disrupts asymmetry division of oocyte and polar body extrusion. Hence, *Tet2* is required for normal meiosis progression by regulating SAC and actin cytoskeleton.

As age also alters DNA methylation and Tet enzymes participate in DNA demethylation, we performed low-input methylation sequencing on MII oocytes to look at potential changes in methylome caused by *Tet2* deficiency and natural aging. Global DNA methylation level increased in *Tet2*^−/−^ oocytes, compared with young WT oocytes, and also slightly increased in natural aging/old WT oocytes, despite no statistical differences (Fig. S6A). In comparison of the differentially methylated genes of *Tet2*^−/−^ with those of natural aging, old WT oocytes, about half of hypomethylated genes in old WT oocytes (total 693 hypomethylated genes) were hypomethylated in *Tet2*^−/−^ oocytes (total 602 hypomethylated genes). About one third of hypermethylated genes in natural aging (total 663 hypermethylated genes) also were hypermethylated (237) after *Tet2* deficiency (total 498 hypermethylated genes) (Fig. S6B). These data suggest that *Tet2*^−/−^ and old WT oocytes (or natural aging oocytes) share 1/3–1/2 DNA methylation modifications. Analysis of genome-wide methylation levels revealed that gene body regions were slightly hypermethylated in young *Tet2*^−/−^ and old WT oocytes, and *Tet2*^−/−^ oocytes had relatively lower methylation levels at TSS and TES, compared with those of young and old WT oocytes (Fig. S6C).

Moreover, in old WT oocytes, hypomethylated genes were involved in aging signaling pathway, and hypermethylated genes enriched in regulation of transcription and actin cytoskeleton signaling pathways (Fig. S6D), implying that these signaling pathways may be down-regulated. Indeed, transcriptome data showed that actin cytoskeleton signaling pathway was declined in old WT oocytes (Fig. S5C). Genes involved in regulation of transcription were hypermethylated in both *Tet2*^−/−^ and old WT oocytes (Fig. S6D), indicating that *Tet2*^−/−^ and old oocytes both possibly negatively regulate transcriptional activity. On the other hand, genes involved in cytoskeleton pathways were hypomethylated and coincidently these genes were highly expressed in *Tet2*^−/−^ oocytes, while they were hypermethylated in old WT oocytes (Fig. S6D). Promoter methylation levels of actin cytoskeleton were reduced in *Tet2*^−/−^ oocytes, but not in old WT oocytes (Fig. S6E). Yet, promoter methylation levels of genes enriched in metabolic process were higher in *Tet2*^−/−^ and old WT oocytes (Fig. S6F). Promoter methylation levels of genes enriched in sister chromatid segregation (GO: 0000819), spindle checkpoint (GO: 0031577), DNA repair (GO: 0006281) and cellular response to DNA damage (GO: 0006974) were higher in young *Tet2*^−/−^ and old WT oocytes compared with young WT oocytes (Fig. S6F). We also analyzed methylation levels at the enhancers of the same genes and showed that the methylation levels at the enhancers generally were higher, but did not show significant changes except for higher levels at DNA repair genes in *Tet2*^−/−^ oocytes than in WT oocytes (Fig. S6G). Overall, *Tet2*-deficient and natural aging oocytes exhibit similarities in methylation modifications at some regions, but differ also in others, for instance, in the methylation of actin cytoskeleton signaling pathway.

Additionally, Tet proteins themselves also have been found to regulate enhancer activity (Lu et al., 2014). We also attempted to look at the potential regulation of gene expression changes using ChIP data reported in ESCs (Hon et al., 2014). *Tet2* deletion resulted in increased 5hmC binding to the gene body. We further analysed the 5hmC binding of genes in actin cytoskeleton and 5hmC binding indeed was increased after *Tet2* knockdown (Fig. S7A), although global 5hmC levels were generally reduced in *Tet2* knockdown compared with WT controls (Fig. S7B). H3K27ac binding signal at these genes enriched in actin cytoskeleton was noticeably increased in *Tet2*^−/−^ oocytes but H3K27me3 showed only slight changes and the binding signal was much lower than that of H3K27ac (Fig. S7C). It appears that increased expression of genes related to actin cytoskeleton coincided with elevated H3K27ac enrichment. But, histone modifications in oocytes and ESCs may be not the same, and these regulations require further investigation.

Together, the whole knockout mouse model demonstrated that *Tet2* deficiency decreases oocyte development and quality, and accelerates age-associated infertility. The *in vitro* embryo developmental delay and incompetence is consistent with reduced embryo development *in vivo*, supporting the accelerated infertility with mouse age. *Tet2* and its mediated DNA methylation changes play critical role in germ cells at various developmental stages. Tet1 has an important role in meiotic gene activation at entry into prophase I in the embryonic ovary and *Tet1* deficiency significantly reduces oocyte numbers and fertility by increased apoptosis (Yamaguchi et al., 2012). We show that effects of *Tet2* on meiosis prophase I appear to be less, relative to the function of *Tet1* in embryonic gonads. Possible compensatory effects of *Tet1* and *Tet3* in the *Tet2*-deficient mice also cannot be excluded. *Tet2* deficiency did not directly elevate apoptosis, and this may allow the defective germ cells with aberrant meiosis progression to survive.

*Tet2* and its role in regulating DNA methylation levels are involved in meiosis, DNA repair, actin cytoskeleton and spindle assembly checkpoint, chromosome alignment and segregation, and thus ploidy of oocytes. Our data suggests that promoter hypermethylation resulting from *Tet2*-deficiency specifically regulates genes in meiosis cell cycle, spindle assembly checkpoint and DNA repair, and the aberrantly increased enhancer activity may contribute to elevated actin cytoskeleton signaling. It is possible that both DNA methylation and enhancer activity by Tet-mediated DNA demethylation regulate specific genes. Indeed, Tet-mediated DNA demethylation mainly occurs at distal regulatory enhancer elements/regions, and enhancers are generally hypomethylated when they are bound by cell type-specific transcription factors (Lu et al., 2014). Hypermethylation can be found at enhancers (H3K4me1 and H3K27ac) of some genes and hypomethylation also can be found at enhancers of other genes depending on their regulatory elements and heterochromatin marks in these regions (Lu et al., 2014). Also, we cannot exclude the possibility that other factors may indirectly affect oocyte quality by *Tet2* deficiency and reduced 5hmC levels in other somatic cells, including bone marrow defects. Nevertheless, the fertility defects in *Tet2*^−/−^ mice mainly result from defective meiotic progression and thus declined quality of oocytes. In future experiments, germline specific conditional knockout of *Tet2* can be created to confirm this observation. These findings broaden our understanding of the role of Tet enzyme in regulating fertility and reproductive aging. Notably, *Tet2* deficiency results in aberrant activation of actin cytoskeleton that impedes asymmetry division of oocytes, suggesting important roles of Tet2 in regulating cell division. Defective asymmetry cell division by *Tet2* mutation or deficiency also may be implicated in somatic stem cell aging.

## Electronic supplementary material

Below is the link to the electronic supplementary material.

Supplementary material 1 (PDF 823 kb)
