# Supplementary material for: Roles of Tet2 in meiosis, fertility and reproductive aging
Source: Protein Cell. 2020 Nov 20;12(7):578–85. doi: 10.1007/s13238-020-00805-8 (PMC8225707; doi:10.1007/s13238-020-00805-8)
Supplement: Supplementary file 1 — Supplementary material 1 (PDF 823 kb) [file 13238_2020_805_MOESM1_ESM.pdf]

Supplemental information

Suppemental Methods
Figures S1-7
Tables S1-2

Supplemental Methods

Tet2 knockout mice.

Tet2 knockout (KO) mice were generated from 129 and C57BL/6J mixed genetic background (Hu et al., 2014). Most of wild type and *Tet2* knockout mice were generated from *Tet2* heterozygotes mating. For fertility analysis, *Tet2* KO females were crossed with WT male at young age. To ensure mating, vaginal plugs were checked daily. All mice in the study were maintained in a mixed 129 and C57BL/6 background. All mouse experiments were carried out in accordance with the guidelines and regulations and approved by the Institutional Animal Care and Use Committee of Nankai University.

Genotyping.

Postnatal mice at two weeks old were genotyped using DNA extracted from their ears. PCR was carried out at 94°C for 2 min, followed by 35 cycles at 94°C for 30 s, 60 °C for 30 s and 72 °C for 1 min. DNA fragments were visualized by agarose gel electrophoresis. The genotyping primers were listed in Table S2.

H&E of ovary and follicle count.

Ovaries were collected from different group mice and fixed by immersion in 4% paraformaldehyde overnight at 4°C, and tissues were embedded with paraffin wax, based on previous methods (Liu et al., 2013). Briefly, serial sections (5 μm) from each ovary were aligned in order on glass microscope slides, stained with hematoxylin and eosin Y, and analyzed for the number of follicles in four different developmental stages in every fifth section with random start in the first section with

ovary. The total number of follicles per ovary was calculated by count of every fifth section throughout the whole ovaries. The follicles were categorized into primordial and primary, secondary and antral, accordingly (Myers et al., 2004). Follicles were classified as primordial and primary if they contained an oocyte surrounded by a single layer of squamous or cuboidal granulosa cells. Secondary follicles were identified as having more than one layer of granulosa cells with no visible antrum. Antral follicles possessed one or two small areas of follicular fluid (antrum) or a single large antral space.

Oocyte collection and *in vitro* fertilization (IVF).

Female mice (WT and *Tet2* KO) from the different groups were superovulated simultaneously by injection of 5 IU pregnant mare's serum gonadotrophin (PMSG), followed 46–48 h later by 5 IU human chorionic gonadotrophin (hCG), to obtain MII oocytes. Oocytes from WT and *Tet2* KO mice were treated with the same operation in the next experiments to minimize the influence of external factors. For *In vitro* maturation (IVM), fully-grown GV oocytes 42-46 h after PMSG injection were collected under a microscope by pricking follicles using insulin syringe in IVM medium. Oocytes were matured *in vitro* by culture in IVM medium at 37 °C (Eppig et al., 2009). IVM medium contains α-MEM (Invitrogen) added with 5% FBS, 0.24 mM sodium pyruvate, 1 IU/ml PMSG and 1.5 IU/ml human chorionic gonadotropin (hCG, Sigma).

For IVF, females were humanely sacrificed and MII oocytes enclosed in cumulus cells collected into Hepes-buffered potassium simplex optimized medium (HKSOM) by releasing from oviduct ampullae 14-16 h after hCG injection. For IVF, spermatozoa were collected from the cauda epididymis of ICR males, capacitated by incubation for 2 h in HTF (Origio), and then incubated with the MII oocytes for 6 h. The zygotes were collected and transferred into human G-1 plus medium (Vitrolife). Embryos that reached the two-cell stage after 24 h culture were cultured in KSOM medium to blastocyst after another 72 h.

Quantitative real-time PCR.

Total RNA was isolated from ovaries using RNeasy mini kit (Qiagen), and subject to cDNA synthesis using Moloney Murine Leukemia Virus Reverse Transcriptase (Invitrogen). PCR reactions were set up in duplicates using the FastStart Universal SYBR Green Master (4913914001, Roche) and run on the Mastercycler® RealPlex2 real time PCR detection system (Eppendorf). At least two parallel samples were run for analysis of each gene. The final PCR reaction volume in 20 μl contained 10 μl SYBR Green PCR Master Mix, 1 μl cDNA template, 2 μl primer mixture and 7 μl water. Thermal cycling was carried out with a 10 min denaturation step at 95 °C, followed by two-step cycles, 15 s at 95 °C and 1min at 60 °C. Each sample was analyzed using GAPDH as the internal control. Primers were listed in Table S1.

PGC purification and RNA-sequencing.

Magnetic Activated Cell Sorting (MACS) assay was used to sort E13.5 PGCs of WT and *Tet2*$^{-/-}$ female mice. Briefly, dissociated gonadal cells were incubated with anti-SSEA1 antibody conjugated with magnetic beads. Cell suspension was washed with PBS supplemented with 0.5% BSA and 2 mM EDTA, an MS column (Miltenyi) was applied to collect SSEA1-positive PGCs. At least three mouse gonads for WT and *Tet2*$^{-/-}$ were purified for RNA extraction as described above. Total RNA from PGCs for RNA-seq was performed by BGI (Beijing Genomics Institute) using BGISEQ-500 platform. The clean reads were aligned to the mouse reference genome using Bowtie2. Prior to differential gene expression analysis, the read counts were calculated by RSEM. All differentially expressed genes were determined by |log2(Fold Change)| > 1 & p-value < 0.001.

Immunofluorescence of spreads, sections and oocytes.

Gonads at genital ridges at E15.5-E18.5 were dissected and prepared for chromosome spreading. The gonads were dissociated by 0.25% trypsin/EDTA (25200072, Life technologies), then the digestion terminated by fetal bovine serum (SH30070.03E, HYCLONE), After centrifugation, cells were suspended in 100 mM

Sucrose and dropped onto slide glasses dipped into fixative (1% paraformaldehyde, 0.15% Triton X-100 and 3 mM dithiothreitol, pH 9.2), followed by slow drying in a humidified box. The slides were washed in water containing 0.4% Photoflow (Kodak), completely dried at room temperature and stored at -20 °C.

Dried slide glasses were washed with 0.1% Triton X-100/PBS (PBST) for 10 min, and incubated with blocking buffer (5% BSA, 1% goat serum and 0.1% Triton X-100 in PBS) for 2 h at room temperature. For the staining with 5hmC antibody, spreads were treated with HCl solution for 20 min at room temperature, followed by washing in PBST. Spreads were then incubated with blocking buffer and primary antibodies at 4 °C overnight, followed by washing with PBST, and incubation with appropriate secondary antibodies for 1 h at room temperature.

For paraffin sections, after deparaffinizing, rehydrating and washing in PBS (pH 7.2–7.4), the sections were subjected to high pressure antigen recovery sequentially in citrate buffer (pH 6.0) for 3 min, incubated with blocking solution (5% goat serum and 0.1% BSA in PBS) for 2 h at room temperature, and then incubated with the diluted primary antibodies overnight at 4 °C. After washing with PBS, sections were incubated with appropriate secondary antibodies (Alexa Fluor® FITC, 488 or 594, Jackson). The sections were then stained with 1 μg/ml Hoechst 33342 for 10 min to reveal nuclei, washed with PBS, mounted in Vectashield (H-1000, Vector Laboratories, Burlingame, CA, US). For frozen section of ovary at PD6, tissues were fixed in 4% paraformaldehyde/PBS for at least 2 h and dehydrated in 30% sucrose overnight at 4 °C, then embedded in OCT compound and cut into 7 μm in thickness. After washing with PBS, sections were permeabilized and blocked as paraffin sections.

Kinetochore associated protein staining was performed as described previously (Liu and Keefe, 2002; Yang et al., 2013). Tubulin, actin and chromatin of oocytes were stained and observed by immunostaining and fluorescence microscopy, as described previously (Allworth and Albertini, 1993). Cumulus cells were removed by pipetting after brief incubation in 0.03% hyaluronidase prepared in HKSOM (Liu and Keefe, 2002). Denuded GV or MII oocytes were fixed and incubated for 30 min at

37 °C in microtubule-stabilizing buffer. Oocytes were washed extensively and blocked for 2 h at room temperature in washing medium (phosphate-buffered saline, supplemented with 0.02% NaN3, 0.01% Triton X-100, 0.2% non-fat dry milk, 2% goat serum, 2% bovine serum albumin and 0.1 M glycine). Afterwards, oocytes were incubated with FITC-α-tubulin (F2168, Sigma) and Phalloidin-iFluor 594 Reagent-cytopainter (ab176757, Abcam) antibodies overnight, washed and counterstained with 0.5 μg/ml DAPI (Roche) in Vectashield (Vector) mounting medium. Fluorescence was detected and imaged using Axio-Imager Z2 Fluorescence Microscope (Carl Zeiss) or Laser Scanning Confocal Microscope (Carl Zeiss, LSM710).

Antibodies	Source	Identifier
Tet2	abcam	Cat#: ab124297
5hmC	active-M	Cat#: 39769
DDX4	abcam	Cat#: ab13840
Oct4	Santa Cruz	Cat#: SC-5279
Mad2	Bethyl	Cat#: A300-301A-T
Cenpe	Abcam	Cat#: ab5093
RAD51	Abcam	Cat#: ab88572
γH2AX	Millipore	Cat#: 05-636
γH2AX	Trevigen	Cat#: 4418-apc-020
SYCP1	Abcam	Cat#: ab15090
SYCP3	Abcam	Cat#: ab97672
SYCP3	Novus	Cat#: NB300-230
MLH1	BD	Cat#: 550838
FITC Goat Anti-Mouse IgG (H+L)	Jackson	Cat#: 115-095-003
Alexa Fluor_ 594 Goat Anti-Rabbit IgG (H+L)	Jackson	Cat#: 111-585-003
Alexa Fluor_ 488 Goat Anti-Rabbit IgG (H+L)	Life	Cat#: A11008
FITC-α-tubulin	Sigma	Cat#: F2168
Phalloidin-iFluor 594 Reagent-cytopainter	Abcam	Cat#: ab176757

The statistics of fluorescence intensity for 5hmC, Mad2, Cenpe and γH2AX were carried by Image J software. Integrated fluorescence intensity of each nucleus of 5hmC or γH2AX and the foci of Mad2 or Cenpe located in kinetochore region were estimated. The threshold was defined using non-specific background fluorescence.

Single-cell isolation and lysis.

Single MII oocyte was resuspended in PBS with 0.1% BSA (A3311-10g, SIGMA), picked up in 1 μl 0.1% BSA using a micropipette with an epT.I.P.S. pipette tip (0030000838, Eppendorf) under a dissecting microscope, and transferred to the bottom of a 200-μl PCR tube (8-strip, nuclease-free, thin-walled PCR tubes with caps, PCR-0208-C, Axygen) containing oligo (dT) primer, Triton X-100, and Recombinant RNase Inhibitor(RRI, 2313A,Takara). Samples were frozen in liquid nitrogen and stored or used immediately.

Reverse transcription.

Frozen or fresh samples were melt on ice and 1 μl deoxy-ribonucleoside triphosphate (R0191, Thermo Scientific) was added into the tubes, vortexed gently, and incubated at 37 °C for 3 min. The cDNAs were then synthesized using template switch oligo (TSO) primer, reverse transcription primer, and SuperScript™ II Reverse Transcriptase (18064071, Thermo Scientific), followed by 15 cycles of PCR using KAPA HotStart ReadyMix (KK2602, KAPA Biosystems) and then purified using Agencount AMPure XP beads (A63881, BECKMAN).

Library construction and sequencing.

RNA-seq libraries were prepared using Smart-seq2 methods as previously described (Picelli et al., 2014). Briefly, the libraries were prepared by using TruePrep DNA Library Prep Kit V2 for Illumina® (TD503-02, Vazyme Biotech) according to the manual instruction. Samples were barcoded during library preparation and multiplex sequenced, with a 150-bp pair-end sequencing strategy on a HiSeq 10x (Illumina).

Single-cell RNA-seq data analysis.

Reads were mapped to mm10 from UCSC genome (http://genome.ucsc.edu/) by Hisat2 (v2.1.0) with default parameters (Kim et al., 2015; Kim et al., 2013). Read counts of each gene annotated in Gencode vM17 were calculated by Featurecounts (Liao et al., 2014), with default parameters. Sum factor normalization was applied with deconvolution of size factors within different batch samples using SCnorm (Bacher et al., 2017). Raw counts were normalized by library size via counts of exon model per million mapped reads (CPM). Principal Component Analysis (PCA) was performed using FactoMineR and factoextra in R package.

Identification of differentially expressed genes.

Dynamic changes of differentially expressed genes (DEGs) between different groups were analyzed using Deseq2 (Love et al., 2014). DEGs were defined only if p-value was < 0.05, and fold change was >1.

GO term gene collection and KEGG pathway analysis.

All GO term gene sets were collected from MGI (Bult et al., 2019) and KEGG pathway term gene sets were collected from KEGG pathway database (Wixon and Kell, 2000). Enrichment results were obtained from clusterprofiler (Yu et al., 2012) and KOBAS (Xie et al., 2011).

DNA methylation library preparation.

DNA methylation libraries of MII oocytes were constructed according to a previously reported method (Smallwood et al., 2014), with minor changes. Ten MII oocytes from three mice (three or more oocytes each) were collected in one PCR tube as a sample, and three Y*Tet2*$^{-/-}$, four YWT, three OWT samples were used as replicates. Bisulfite conversion was performed on cell lysates with the following modifications: incubation at 98°C for 10 min and 64°C for 120 min. DNA was eluted in 10 mM Tris-Cl (pH 8.5) and combined with 10 mM dNTPs, 5 μM BioPEA_N4_37 (5′-biotin-ACACTCTTTCCCTACACGACGCTCTTCCGATCTNNN N-3′), 10 x

NEBuffer 2 (E7645S, NEB) before incubation at 95°C for 5 min followed by 4°C pause. 75 U of Klenow Fragment (M0212M, NEB) was added and the samples incubated at 4°C for 5 min, +1°C/15 s to 37°C, 37°C for 30 min. Samples were incubated at 95°C for 1 min and transferred immediately to ice prior to addition of fresh 1 mM dNTPs, 10 nM BioPEA_N4_37, 10 x NEBuffer 2, and 75 U Klenow Fragment in 2.75 μl total. The samples were incubated at 4°C for 5 min, +1°C/15 s to 37°C for 30 min. This random priming and extension were repeated for further 3 times (5 rounds in total). Samples were then incubated with 40 U exonuclease I (M0293V, NEB) for 1 h at 37°C before DNA was purified using 1 × Agencourt Ampure XP beads (A63881, Beckman) according to the manufacturer's guidelines. Samples were eluted in 10 mM Tris-Cl (pH 8.5) and incubated with washed M-280 Streptavidin Dynabeads (65001, Life Technologies) for 30 min with rotation at room temperature. Beads were washed twice with 0.1 N NaOH, and twice with 10 mM Tris-Cl (pH 8.5) and resuspended in 48 μl of 10 mM dNTPs, 10 x NEBuffer 2, 10 μM Primer 2.0 (5'-GTGACTGGAGTTCAGACGTGTGCTCTTCCGATCTNNNN-3'). Samples were incubated at 95°C for 45 s and transferred immediately to ice before addition of 100 U Klenow Fragment and incubation at 4°C for 5 min, +1°C/15 s to 37°C, and 37°C for 90 min. Beads were washed with 10 mM Tris-Cl (pH 8.5) and resuspended in 50 μl of 1 U KAPA HiFi HotStart DNA Polymerase (KK2801, KAPA Biosystems), 10 uM Primer 1.0 (5'-AATGATACGGCGACCACCGAGATCTACACTCTTTCCCTACACGACGCTCTTCCGATCT-3'), 10 uM Index. Libraries were then amplified by PCR as follows: 98°C 45 s, 8 repeats of (98°C 15 s, 65°C 30 s, 72°C 30 s), 72°C 1 min, and 4°C hold. Amplified libraries were purified using 0.8 × Agencourt Ampure XP beads, according to the manufacturer's guidelines. Samples were eluted in another 27 μl of 1 U KAPA HiFi HotStart DNA Polymerase (KK2801, KAPA Biosystems), 10 μM Primer 1.0 forward primer, 10 μM Index and amplified by PCR for further 8 repeats. Amplified libraries were assessed for quality and quantity using High-Sensitivity DNA chips on the Agilent Bioanalyser. DNA methylation libraries were sequenced by Annoroad using Hiseq 4000 platform.

Sequencing data processing.

Sequencing reads were trimmed to remove the low-quality reads and reads containing adapters. Sequencing reads were trimmed with default setting of Trimmomatic software (Bolger et al., 2014), to remove the adapters and low-quality reads. Trimmed reads were aligned to mouse reference mm10 by using Bismark (v12.5) (Krueger and Andrews, 2011). PCR duplications were removed with Picard (http://broadinstitute.github.io/picard/) and the overlapped regions in uniquely mapped paired reads were clipped with clipOverlap function of BamUtil (http://genome.sph. umich.edu/wiki/BamUtil: clipOverlap). CpG and non-CpG methylation levels were extracted with mpileup function of Samtools (v0.1.19) (Li et al., 2009). Strands were merged to calculate the CpG methylation level per site. Promoters are defined as the regions 1 kb upstream and 1 kb downstream from the transcriptional start sites (TSSs) of mouse Refseq transcripts (mm10). Only promoters containing at least 5 CpGs and covered by at least 10 reads were considered for further analysis. The methylation level of each promoter was calculated as average methylation level of all CpG sites in this promoter. Differentially methylated genes are defined as methylation level differences greater than 0.2 and FDR less than 0.05. These *Tet2* deficiency-specific and aging-specific highly/lowly methylated promoters were subjected to GO analysis. GO analysis of genes with differentially methylated promoters was performed using DAVID 6.8. GO terms with p-value <0.05 were considered statistically significant.

Processing ChIP-seq data.

ChIP-seq data were downloaded from GEO datasets and then reads were aligned to the mouse genome (mm10, UCSC version) using bowtie2 (version 2.2.3) with default parameters (Langdon, 2015). ChIP-seq peaks were visualized using IGV software (Thorvaldsdottir et al., 2013). Bedtools (Quinlan and Hall, 2010) was used to obtain the average signal of selected pathway or GO items. R package including ggplot2, pheatmap and Rtsne were used.

Statistical analysis.

Data were analyzed by student’s t-test, $\chi 2$ test or fisher's exact test for paired comparison, or by Analysis of Variance (ANOVA) and means compared by Fisher’s protected least-significant difference (PLSD) for multiple comparisons using the StatView from SAS Institute and the graphs made by GraphPad Prism 7 (GraphPad Software, San Diego, CA). The data were considered significant when $P < 0.05$ (*), 0.01(**) or 0.001(***).

Data Availability.

RNA-seq and DNA methylation data have been deposited in the GEO database under the accession number GSE142163 and GSE145547, respectively.

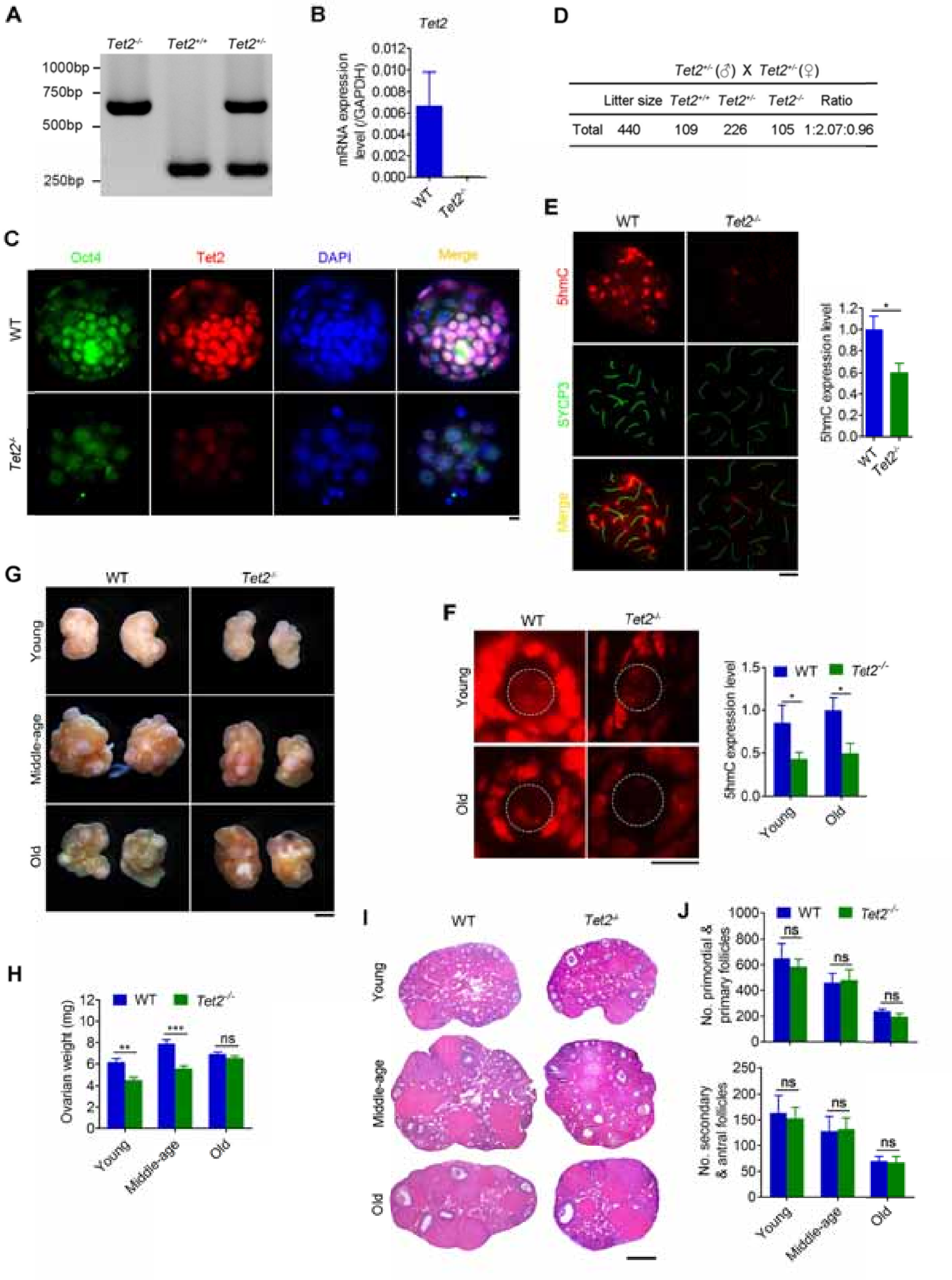

Figure S1. Confirmation of loss of Tet2 enzyme and reduced 5hmC level in female mice.

(A) PCR genotyping of *Tet2*-deficient mice. Primer sequences are listed in Table S2.

(B) *Tet2* mRNA expression level in ovaries of young mice by qPCR analysis.

(C) Tet2 protein expression level in E3.5 blastocysts by immunofluorescence. Scale bar, 10 μm.

(D) Table summarizing the litter size and Mendelian ratio of *Tet2*$^{-/-}$ mice.

(E) Representative images of pachytene meiocytes co-stained with SYCP3 and 5hmC antibodies (left). 5hmC expression level in pachytene meiocytes between WT and *Tet2*$^{-/-}$ mice (right). n>80 cells counted in each group. Scale bar, 10 μm.

(F) Representative images of primordial follicles stained with 5hmC antibody. Dashed line indicates the primordial oocyte (left). Scale bar, 10 μm. 5hmC level in primordial oocytes (right panel). n≥12 oocytes counted in each group. Data represents mean ± SEM. *P <0.05.

(G) Morphology of ovary from three different age groups (Young, Middle-age and Old). Scale bar, 1 mm.

(H) Average weight per ovary from WT and *Tet2*$^{-/-}$ mice. n=20 for each group.

(I) Representative images of haematoxylin and eosin (H&E) staining of ovarian sections. Scale bar, 500 μm.

(J) Number of primordial and primary follicles, or secondary and antral follicles from WT and *Tet2*$^{-/-}$ mice. n=4-6.

The bars indicate mean ± SEM. *P < 0.05; **P < 0.01; ***P < 0.001; ns, not significantly different.

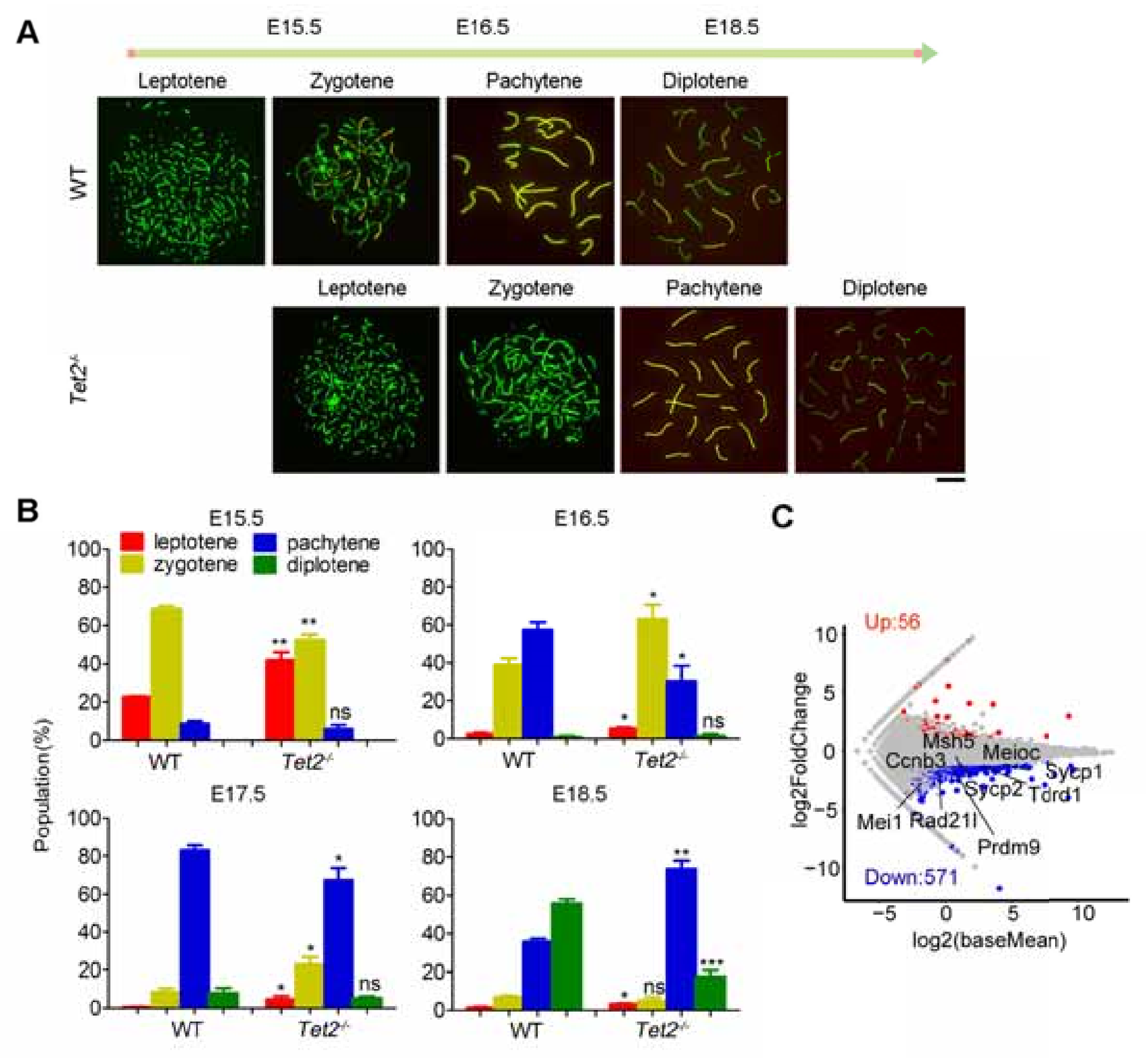

Figure S2. Loss of *Tet2* results in delay of meiosis progression in fetal gonad.

(A) Representative images of meiocytes stained with antibodies against SYCP1 (red) and SYCP3 (green) from wild-type (WT) and *Tet2*$^{-/-}$ mice. Scale bar, 10 µm. Orange, SYCP3 merged with SYCP1.

(B) Proportion of the four stages of prophase I in E15.5, E16.5, E17.5 and E18.5 meiocyte populations of WT and *Tet2*$^{-/-}$ gonads. n=3-6. Mean ± SEM. *P<0.05; **P < 0.01. ***P < 0.001.

(C) Scatter plot comparing transcriptome of WT and *Tet2*$^{-/-}$ E13.5 female meiocytes. 56 (in red) and 571 (in blue) genes are up- or downregulated respectively (log2Foldchange > 1 or < -1, p < 0.05). Examples of downregulated genes involved in meiotic cell cycle are highlighted.

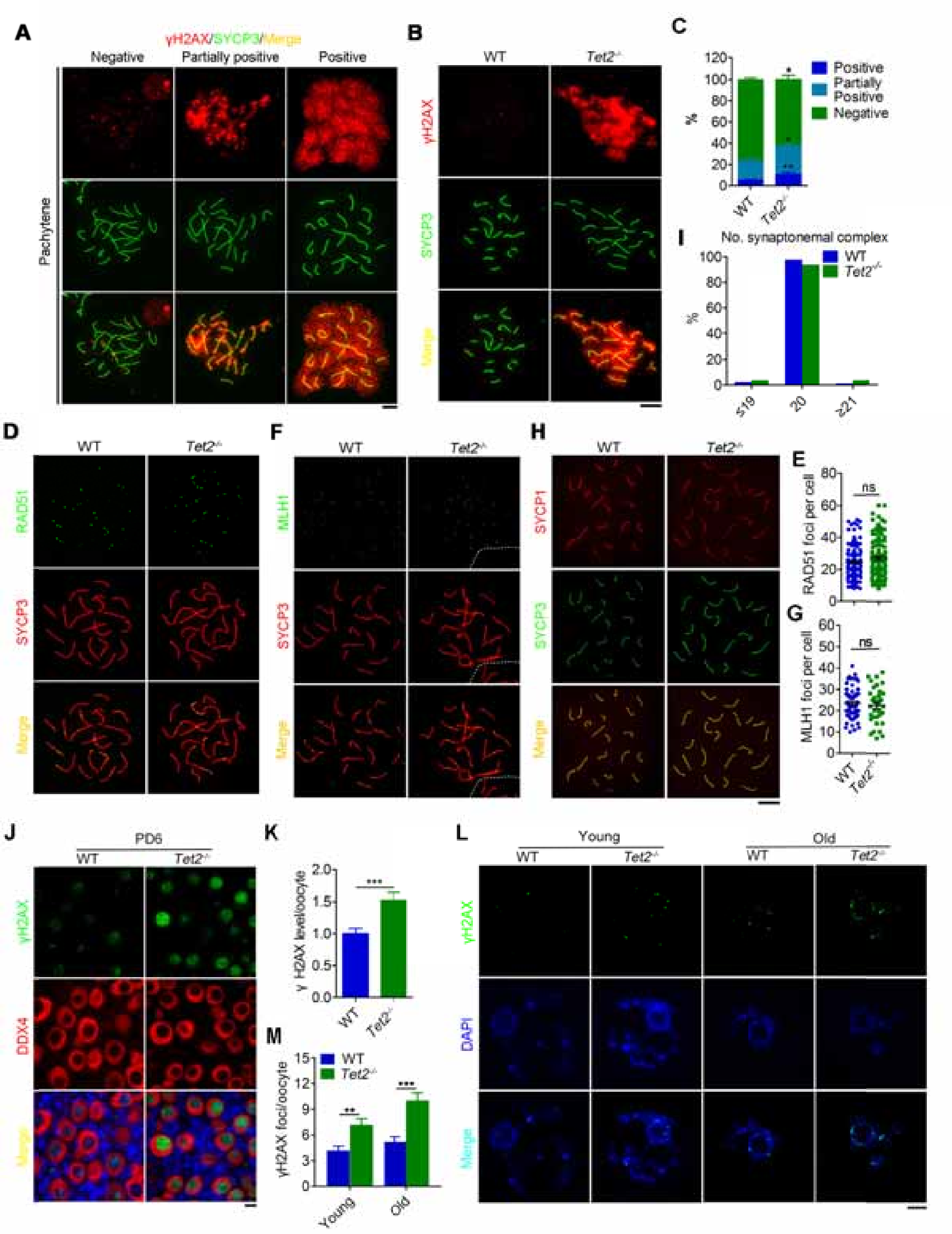

Figure S3. *Tet2$^{-/-}$* oocytes accumulate DNA damage.

(A) Representative images of pachytene meiocytes classified based on the level of γH2AX accumulation co-stained with SYCP3 and γH2AX antibodies. Scale bar, 10 μm.

(B) Representative images from WT and *Tet2$^{-/-}$* pachytene meiocytes co-stained

with SYCP3 and γH2AX antibodies. Scale bar, 10 μm.

(C) Proportion of oocytes showing pattern of γH2AX in pachytene meiocytes. n≥3 mice and more than 100 meiocytes counted for each group.

(D,E) Representative images from wild-type (WT) and *Tet2*$^{-/-}$ in pachytene meiocytes co-stained with SYCP3 and RAD51 antibodies (D). Number of RAD51 foci in pachytene meiocytes (E). n≥3 mice and more than 100 meiocytes counted for each group.

(F,G) Representative images from WT and *Tet2*$^{-/-}$ in pachytene meiocytes co-stained with SYCP3 and MLH1 antibodies (F). Number of MLH1 foci in each pachytene stage meiocyte (G). n=3 mice and more than 40 meiocytes counted for each group.

(H,I) Representative images from WT and *Tet2*$^{-/-}$ in pachytene meiocytes co-stained with SYCP3 and SYCP1 antibodies (H). Distribution in the number of synaptonemal complex of WT and *Tet2*$^{-/-}$ pachytene meiocytes (I). n=3 mice and about 50 meiocytes was counted for each group. Scale bar, 10 μm.

(J,K) Representative images of primordial follicles co-stained with γH2AX and DDX4 antibodies from ovarian sections at postnatal day 6 (PD6) (J). Comparison of γH2AX intensity per oocyte between WT and *Tet2*$^{-/-}$ (K). Scale bar, 10 μm.

(L,M) Representative images of germinal vesicle (GV) oocytes stained with γH2AX (L) and comparison of γH2AX foci per oocyte between WT and *Tet2*$^{-/-}$ (M). Scale bar, 10 μm. Mean ± SEM. *P < 0.05; **P < 0.01; ***P < 0.001.

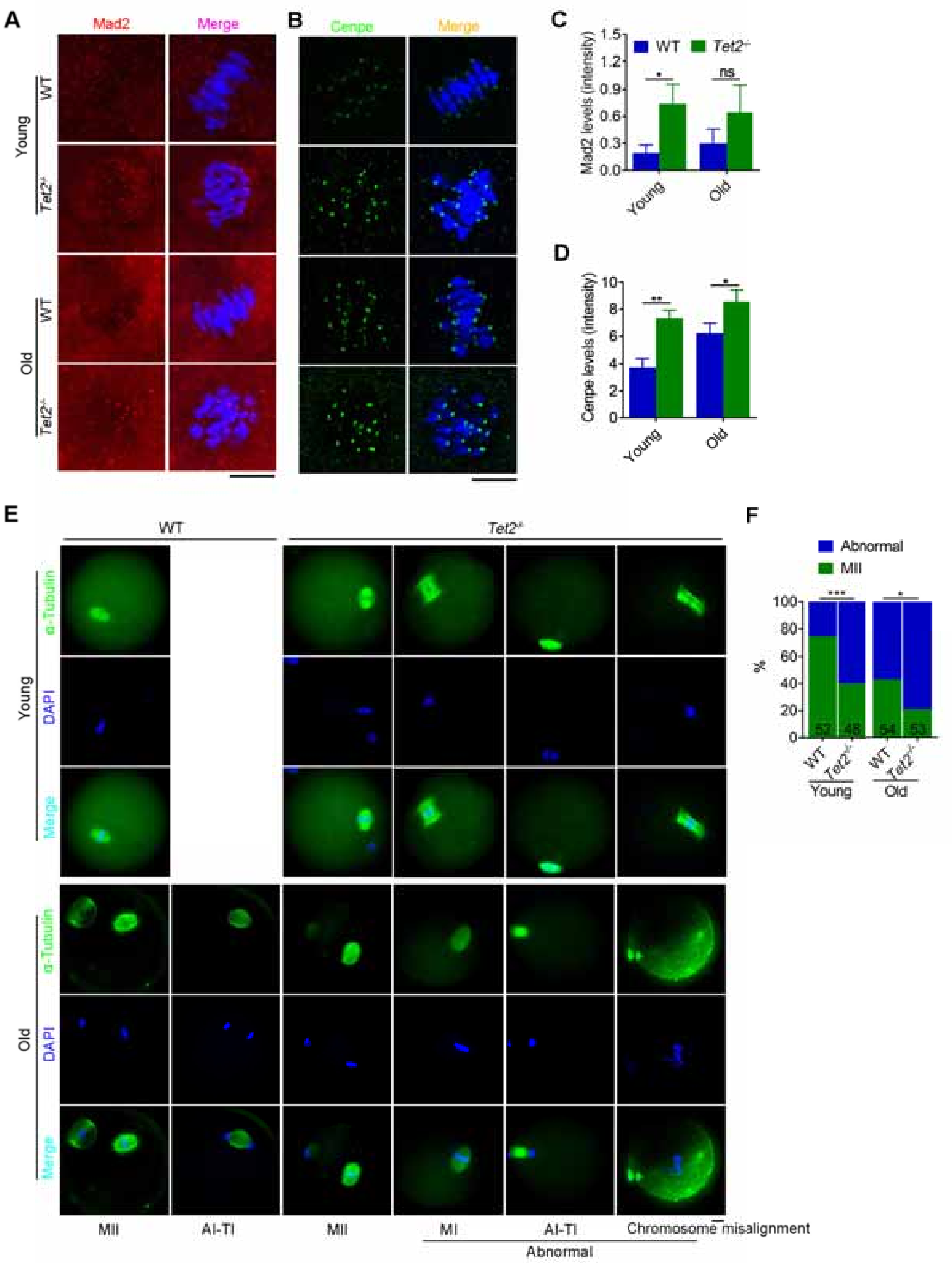

Figure S4. *Tet2* deficiency delays meiosis I progression shown by Mad2 and Cenpe immunofluorescence and increases abnormal oocytes.

(A) Representative images of MI oocytes stained with Mad2 antibody.

(B) Representative images of MI oocytes stained with Cenpe antibody. Scale bar,

10 μm.

(C) Comparison of Mad2 immunofluorescence intensity between WT and *Tet2*$^{-/-}$ oocytes.

(D) Comparison of Cenpe immunofluorescence intensity between WT and *Tet2*$^{-/-}$ oocytes.

(E) Representative images of ovulated oocytes at different stages by spindle organization and chromosome separation. MI, metaphase I; AI-TI, anaphase I and telophase I; MII, metaphase II. Scale bar, 10 μm.

(F) Distribution of normal and abnormal oocytes. Normal oocytes refer to those at MII stage with proper chromosome alignment at the intact spindle. Abnormal oocytes refer to those at MI, AI, TI and aberrant chromosome alignment and disrupted spindle. n=3 mice for young age and n=4-5 mice for old age. Statistical significance is analyzed by $\chi 2$ test.

Mean ± SEM. *P < 0.05; **P < 0.01. ***P < 0.001.

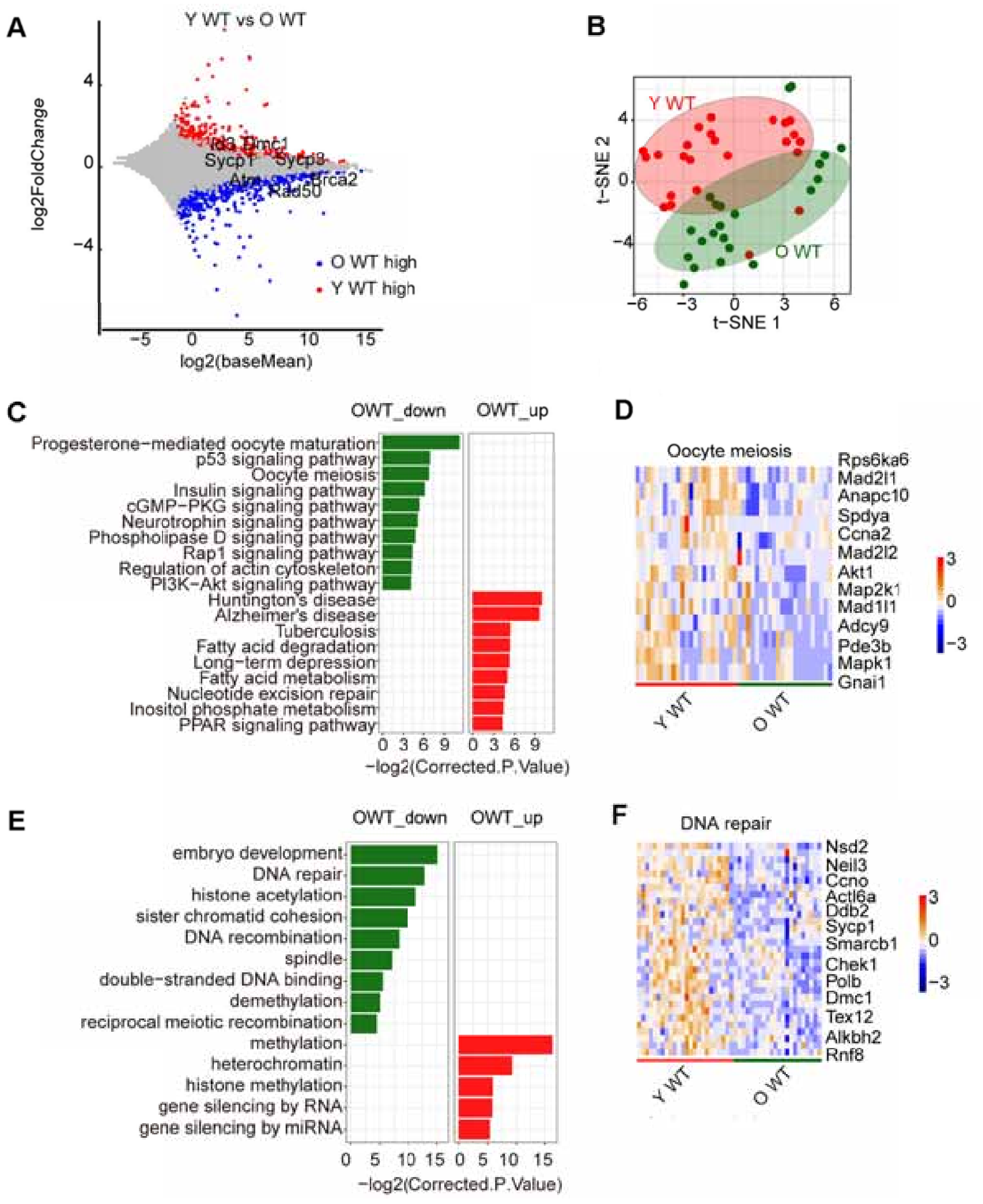

Figure S5. Differential gene expression of young and old WT oocytes.

(A) Scatter-plots showing gene expression between young WT (n=24 oocytes from 3 mice) and old WT oocytes (n=22 oocytes) from three mice.

(B) t-distributed Stochastic Neighbor Embedding (t-SNE) analysis of young and old WT oocytes using the CPM for all genes.

(C) KEGG enrichment results showing genes downregulated and upregulated in old WT oocytes. X-axis represents the corrected p-value (FDR) by Benjamini and

Hochberg.

(D) Pheatmap showing genes enriched in oocyte meiosis, downregulated in old WT oocytes.

(E) GO enrichment results showing that genes downregulated in old WT oocyte are enriched in demethylation, double-strand DNA binding, spindle, DNA recombination, sister chromatid cohesion, histone acetylation, DNA repair, and embryo development. Genes upregulated are enriched in heterochromatin and methylation. X-axis represents the corrected p-value (FDR) by Benjamini and Hochberg.

(F) Pheatmap showing genes enriched in DNA repair, downregulated in old WT oocytes.

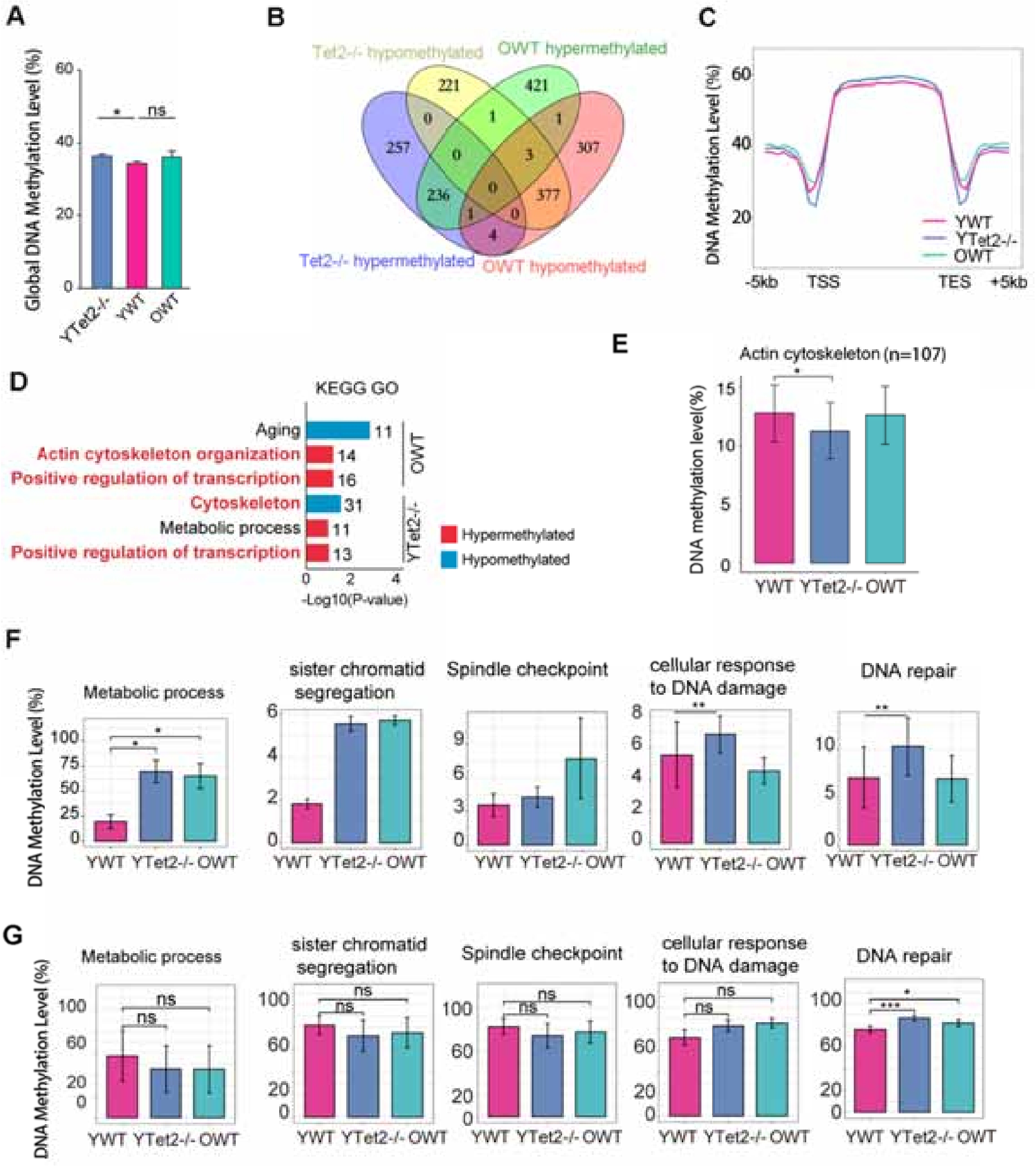

Figure S6. Methylation of WT and *Tet2*$^{-/-}$ oocytes.

(A) Global DNA methylation level of young WT oocytes (n=40) from 4 mice and *Tet2*$^{-/-}$(n=30 oocytes) from 3 mice, and old WT oocytes (n=30) from 3 mice. *P< 0.05.

(B) Venn diagram of differentially methylated regions from young *Tet2*$^{-/-}$ and old WT oocytes, compared with young WT oocytes. Differentially methylated regions are defined as methylation level differences greater than 0.2 and FDR less than 0.05.

(C) The average DNA methylation levels (CpG sites) along the gene bodies, 5 kb upstream of the transcription start sites (TSSs) and 5 kb downstream of the transcription end sites (TES) of all the RefSeq genes across different samples.

(D) GO enrichment of differentially methylated regions in old WT and young *Tet2*$^{-/-}$ oocytes, compared with those of young WT oocytes. The number at the right-hand side of the bar indicates the number of genes.

(E) Barplot showing the promoter methylation levels of genes enriched in actin cytoskeleton pathway. Kruskal-Wallis test was used to compare between multiple groups. Data are shown as mean ± SEM. *P< 0.05.

(F) Promoter methylation levels of genes enriched in metabolic process, sister chromatid segregation, spindle checkpoint, DNA repair and cellular response to DNA damage stimulus. Kruskal-Wallis test was used to compare between multiple groups. Data are shown as mean ± SEM. *P< 0.05, **P< 0.01, ns, no significant difference.

(G) Enhancer methylation levels of genes enriched in metabolic process, sister chromatid segregation, spindle checkpoint, DNA repair and cellular response to DNA damage stimulus. Kruskal-Wallis test was used to compare between multiple groups. Data are shown as mean ± SEM. *P< 0.05, **P< 0.01, ns, no significant difference.

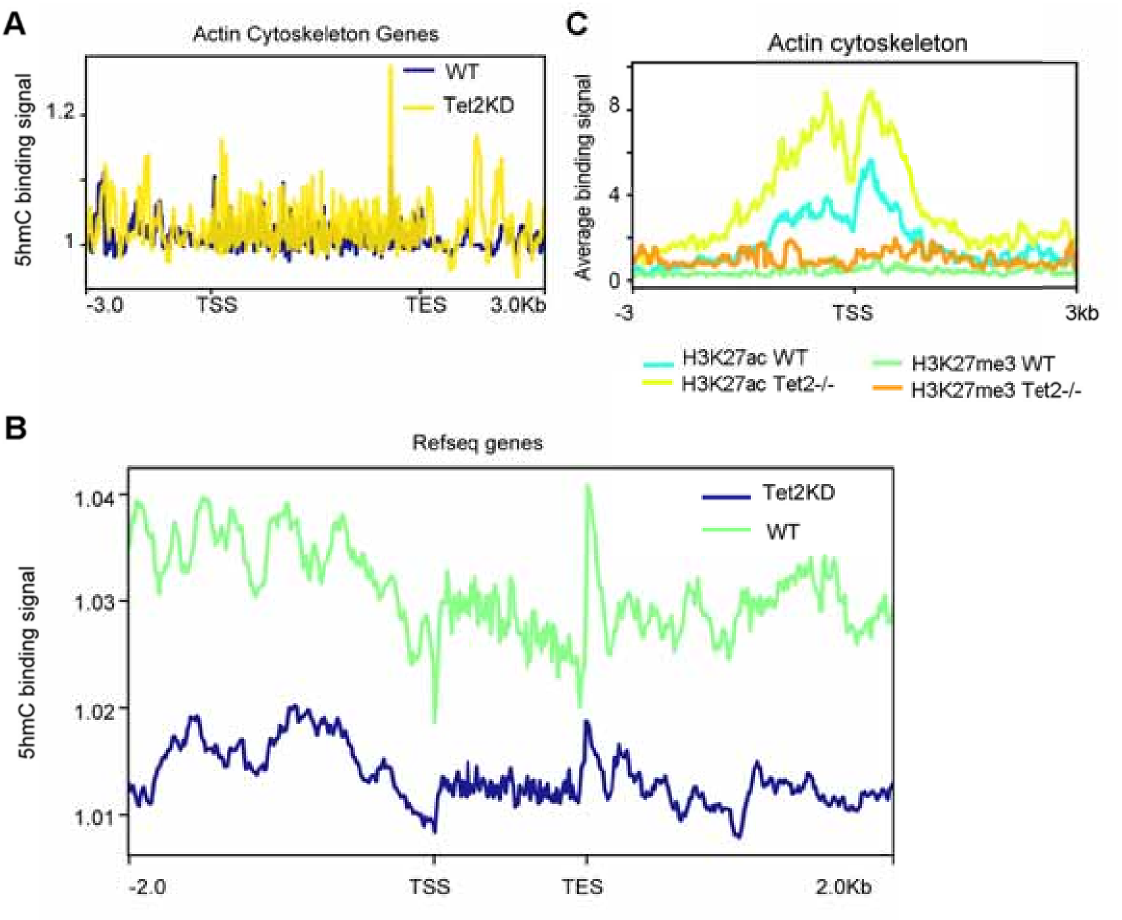

Figure S7. Additional epigenetic regulation of Tet2.

(A) 5hmC binding signal of genes enriched in actin cytoskeleton, signal pathway to which genes are up-regulated in young *Tet2*$^{-/-}$ oocytes. Data was obtained from GEO datasets (GSE50201(Huang et al., 2014)).

(B) Comparison of global 5hmC levels in *Tet2* KD and WT ESCs. Data was obtained from GEO datasets (GSE50201).

(C) H3K27ac and H3K27me3 binding signal of genes enriched in actin cytoskeleton, a signal pathway to which genes are up-regulated in young *Tet2*$^{-/-}$ oocytes. Data was obtained from GEO datasets (GSE48519(Hon et al., 2014))

Table S1. Primers for real-time qPCR analysis

Genes	Sequence
Tet2-Foward	TGTTGTTGTCAGGGTGAGAATC
Tet2-Reverse	TCTTGCTTCTGGCAAACTTACA
Gapdh-Foward	TCAACAGCAACTCCCACTCTTCCA
Gapdh-Reverse	ACCACCCTGTTGCTGTAGCCGTAT

Table S2. Primers for genotyping

Genes	Sequence
Tet2-Gf1	AGTTCACCCTTCTCATGTGGATACT
Tet2-Gr1	CTCTTTACCATACTTGATTGGCTCT
Tet2-Gr4-6	AGTCCTTGGTCATCAGGAACTCTA

References

Allworth, A.E., and Albertini, D.F. (1993). Meiotic maturation in cultured bovine oocytes is accompanied by remodeling of the cumulus cell cytoskeleton. Dev Biol *158*, 101-112.

Bacher, R., Chu, L.F., Leng, N., Gasch, A.P., Thomson, J.A., Stewart, R.M., Newton, M., and Kendziorski, C. (2017). SCnorm: robust normalization of single-cell RNA-seq data. Nat Methods *14*, 584-586.

Bolger, A.M., Lohse, M., and Usadel, B. (2014). Trimmomatic: a flexible trimmer for Illumina sequence data. Bioinformatics *30*, 2114-2120.

Bult, C.J., Blake, J.A., Smith, C.L., Kadin, J.A., Richardson, J.E., and Mouse Genome Database, G. (2019). Mouse Genome Database (MGD) 2019. Nucleic acids research *47*, D801-D806.

Eppig, J.J., O'Brien, M.J., Wigglesworth, K., Nicholson, A., Zhang, W., and King, B.A. (2009). Effect of in vitro maturation of mouse oocytes on the health and lifespan of adult offspring. Hum Reprod *24*, 922-928.

Hon, G.C., Song, C.X., Du, T., Jin, F., Selvaraj, S., Lee, A.Y., Yen, C.A., Ye, Z., Mao, S.Q., Wang, B.A.*, et al.* (2014). 5mC oxidation by Tet2 modulates enhancer activity and timing of transcriptome reprogramming during differentiation. Mol Cell *56*, 286-297.

Hu, X., Zhang, L., Mao, S.Q., Li, Z., Chen, J., Zhang, R.R., Wu, H.P., Gao, J., Guo, F., Liu, W.*, et al.* (2014). Tet and TDG mediate DNA demethylation essential for mesenchymal-to-epithelial transition in somatic cell reprogramming. Cell Stem Cell *14*, 512-522.

Huang, Y., Chavez, L., Chang, X., Wang, X., Pastor, W.A., Kang, J., Zepeda-Martinez, J.A., Pape, U.J., Jacobsen, S.E., Peters, B.*, et al.* (2014). Distinct roles of the methylcytosine oxidases Tet1 and Tet2 in mouse embryonic stem cells. Proc Natl Acad Sci U S A *111*, 1361-1366.

Kim, D., Langmead, B., and Salzberg, S.L. (2015). HISAT: a fast spliced aligner with low memory requirements. Nat Methods *12*, 357-360.

Kim, D., Pertea, G., Trapnell, C., Pimentel, H., Kelley, R., and Salzberg, S.L. (2013).

TopHat2: accurate alignment of transcriptomes in the presence of insertions, deletions and gene fusions. Genome Biology *14*.

Krueger, F., and Andrews, S.R. (2011). Bismark: a flexible aligner and methylation caller for Bisulfite-Seq applications. Bioinformatics *27*, 1571-1572.

Langdon, W.B. (2015). Performance of genetic programming optimised Bowtie2 on genome comparison and analytic testing (GCAT) benchmarks. BioData mining *8*, 1.

Li, H., Handsaker, B., Wysoker, A., Fennell, T., Ruan, J., Homer, N., Marth, G., Abecasis, G., Durbin, R., and Genome Project Data Processing, S. (2009). The Sequence Alignment/Map format and SAMtools. Bioinformatics *25*, 2078-2079.

Liao, Y., Smyth, G.K., and Shi, W. (2014). featureCounts: an efficient general purpose program for assigning sequence reads to genomic features. Bioinformatics *30*, 923-930.

Liu, L., and Keefe, D.L. (2002). Ageing-associated aberration in meiosis of oocytes from senescence-accelerated mice. Hum Reprod *17*, 2678-2685.

Liu, M., Yin, Y., Ye, X., Zeng, M., Zhao, Q., Keefe, D.L., and Liu, L. (2013). Resveratrol protects against age-associated infertility in mice. Hum Reprod *28*, 707-717.

Love, M.I., Huber, W., and Anders, S. (2014). Moderated estimation of fold change and dispersion for RNA-seq data with DESeq2. Genome Biol *15*, 550.

Myers, M., Britt, K.L., Wreford, N.G., Ebling, F.J., and Kerr, J.B. (2004). Methods for quantifying follicular numbers within the mouse ovary. Reproduction *127*, 569-580.

Picelli, S., Faridani, O.R., Bjorklund, A.K., Winberg, G., Sagasser, S., and Sandberg, R. (2014). Full-length RNA-seq from single cells using Smart-seq2. Nat Protoc *9*, 171-181.

Quinlan, A.R., and Hall, I.M. (2010). BEDTools: a flexible suite of utilities for comparing genomic features. Bioinformatics *26*, 841-842.

Smallwood, S.A., Lee, H.J., Angermueller, C., Krueger, F., Saadeh, H., Peat, J., Andrews, S.R., Stegle, O., Reik, W., and Kelsey, G. (2014). Single-cell

genome-wide bisulfite sequencing for assessing epigenetic heterogeneity. Nat Methods *11*, 817-820.

Thorvaldsdottir, H., Robinson, J.T., and Mesirov, J.P. (2013). Integrative Genomics Viewer (IGV): high-performance genomics data visualization and exploration. Briefings in bioinformatics *14*, 178-192.

Wixon, J., and Kell, D. (2000). The Kyoto encyclopedia of genes and genomes--KEGG. Yeast *17*, 48-55.

Xie, C., Mao, X., Huang, J., Ding, Y., Wu, J., Dong, S., Kong, L., Gao, G., Li, C.Y., and Wei, L. (2011). KOBAS 2.0: a web server for annotation and identification of enriched pathways and diseases. Nucleic acids research *39*, W316-322.

Yang, K.T., Lin, Y.N., Li, S.K., and Tang, T.K. (2013). Studying the roles of Aurora-C kinase during meiosis in mouse oocytes. Methods Mol Biol *957*, 189-202.

Yu, G., Wang, L.G., Han, Y., and He, Q.Y. (2012). clusterProfiler: an R package for comparing biological themes among gene clusters. Omics : a journal of integrative biology *16*, 284-287.
